# An In Vitro Evaluation of the Effects of Air-Polishing Powders on Sound and Demineralised Enamel

**DOI:** 10.3390/ma16134811

**Published:** 2023-07-04

**Authors:** Elton Guma, Stavros Kiliaridis, Susanne S. Scherrer, Gregory S. Antonarakis

**Affiliations:** 1 Division of Orthodontics, University Clinics of Dental Medicine, University of Geneva, 1205 Geneva, Switzerland; elton.guma@etu.unige.ch (E.G.); stavros.kiliaridis@unige.ch (S.K.); 2 Department of Orthodontics and Dentofacial Orthopedics, University of Bern, 3012 Bern, Switzerland; 3 Division of Fixed Prosthetics and Biomaterials, University Clinics of Dental Medicine, University of Geneva, 1205 Geneva, Switzerland; susanne.scherrer@unige.ch

**Keywords:** enamel, demineralised enamel, air polishing, erythritol, bicarbonate, white spot lesions, orthodontics, surface roughness

## Abstract

Aim: To evaluate the effects of two air-polishing powders, during orthodontic treatment, on the surface roughness of sound and demineralised enamel. Materials and methods: Forty-two caries-free human molars were collected, and the enamel surfaces were flattened and polished. Teeth were assigned to two groups (*n* = 21 each), a sound- and a demineralised-enamel group (subjected to pH-cycling over 2 weeks to create artificially induced white spot-like lesions). Within each group, teeth were further assigned to three groups (*n* = 7 each), air polished with either sodium bicarbonate, erythritol, or a negative control (water). Each sample was treated for 5 and 150 s. The average surface roughness (Ra) for each sample was measured using white-light-sensor profilometry. Results: On sound enamel, the Ra was roughly 0.17 ± 0.07 μm. After 150 s of air polishing, the Ra increased with erythritol (by 0.28 μm), and even more so with bicarbonate treatment (by 0.68 μm) (*p* < 0.01). On demineralised enamel, the Ra was roughly 0.79 ± 0.56 μm. The Ra increased after 5 s of air-polishing treatment similarly with erythritol and bicarbonate powders (by 1.03 μm and 1.04 μm, respectively) (*p* = 0.025), and even more after 150 s (by 2.48 μm and 2.49 μm, respectively) (*p* < 0.001). Conclusions: On white spot lesions, one should be aware that enamel surface roughness will increase with both erythritol and bicarbonate air-polishing powders, especially with longer exposure times.

## 1. Introduction

During orthodontic treatment, the placement of fixed appliances (brackets, bands) impairs oral hygiene, resulting in more plaque accumulation, gingival inflammation, and bleeding [1]. Early dental decay or demineralised lesions (also known as white spot lesions) developing around the bracket margins are still one of the major complications of orthodontic treatment [2,3].

Daily oral hygiene measures have a limited capacity in remove newly formed bacterial deposits, and thus regular professional tooth cleaning is recommended, especially during active orthodontic treatment. Ultrasonic debridement, manual scaling, rubber-cup polishing with prophylactic paste, and air polishing are often-used approaches for oral prophylaxis, to remove plaque and staining during orthodontic treatment.

Air polishing has recently gained popularity. This is performed by the propulsion of air-polishing paste particles (with a mixture of compressed air and water) through a handpiece nozzle against the tooth surface, in an attempt to remove any dental plaque, biofilm, and soft deposits (staining) that are present [4]. Air-polishing devices (APDs) have been suggested as an alternative for periodontal maintenance [5,6] and are used on the tooth enamel of calculus-free surfaces or after existing calculus has been removed with the use of more aggressive types of instrumentation [7,8].

Several advantages have been attributed to APDs, with claims stating that they are highly effective, easy to use, and time-efficient, and that they improve access to hardly accessible tooth surfaces, lead to less operator fatigue, reduce post-operative patient discomfort and sensitivity, and are associated with high patient acceptance rates [6,9]. When using APDs, the most critical item in the air-polishing armamentarium is the polishing powder itself, which is also important in bacterial reduction [10,11].

It is important, therefore, for the powder used to be the least damaging as possible to the enamel surface, while maintaining its prophylactic properties. The enamel surface must remain intact and smooth, as must the surrounding soft tissue structures, and the powder used should be physiologically compatible with the oral tissues and digestive system [12].

Specially processed (salt) sodium bicarbonate (NaHCO_3_) was one of the first air-polishing powders used, and studies have confirmed its safety and efficacy when used supra-gingivally, when compared with conventional scaling and rubber-cup polishing, despite its abrasive nature on the root surface and some restorative materials [5]. In recent years however, with the intention to eliminate the abrasive nature of larger-particle polishing powders, alternative resorbable powders with low abrasiveness, such as reduced particle size sodium-bicarbonate, glycine, and erythritol, have been introduced [13,14]. Glycine, for example, has been found to be minimally abrasive on root dentine [15] and cementum [16].

Erythritol is the latest low-abrasive powder used in association with chlorhexidine, introduced by Electro Medical System (EMS, Nyon, Switzerland). Erythritol is a chemically neutral water-soluble 4-carbon sugar alcohol (polyol) with non-toxicological effects, used primarily as a low-calorie sweetener, but also found widely in several natural foods and endogenously in tissues and body fluids [17]. It has been found to be non-cariogenic [18], more effective than the traditional polishing on supragingival biofilm removal [19], and has even been suggested to have a possible caries-limiting potential by inhibiting the growth of several strains of mutans streptococci [20].

Erythritol polishing powders, in a preclinical characterisation study, reveal smaller particle sizes and a slightly lower abrasiveness on dental root tissues when compared with glycine air-polishing powders [8]. Moreover, when compared with sodium bicarbonate and glycine powder in vitro, erythritol induces the lowest volume loss and defect depth on dentine, and produces the smoothest surface [21]. It seems, therefore, that erythritol powders show a safer profile than other air-polishing powders. Moreover, erythritol may represent a promising modality for the removal of biofilm [8] during fixed orthodontic treatment.

With regard to orthodontic treatment, not much research has been conducted looking into the effects of air polishing. Cleaning a tooth surface with an air-polishing powder prior to bracket placement on the enamel surface can influence the bond strength but with no detrimental effects [22].

For patients in orthodontic treatment, it has been recommended to use erythritol air-polishing powders (reported particle size of 14 μm) and glycine powders (reported particle size of 25 μm) for prophylaxis in patients wearing fixed orthodontic appliances, but this recommendation comes from the company that produces the APDs [23]. One potential concern, however, may be related to its repeated use, which may potentially contribute to the removal of tooth substance or a change in the surface roughness of the enamel. In a recent study, Ratzka et al. [24], considering 25 air-polishing treatments on bovine high-gloss polished sound enamel, found that the regular use of sodium bicarbonate and erythritol powder during a simulated fixed appliance therapy led to a highly significant increase in surface roughness, in which sodium bicarbonate was significantly more abrasive than erythritol.

Other technical parameters, such as the mode of clinical application, including distance, angulation, and treatment time, may affect the potential changes to the enamel surface, in addition to the choice of air-polishing powder itself, but not much is known about the effect of the repeated use of air-polishing powders during orthodontic treatment on the human enamel surface. In addition, little is known about the influence of air-powder polishing on demineralised enamel lesions, which are common occurrences during orthodontic treatment that change the enamel surface structure.

The aim of the present study was to evaluate the effects of repeated air polishing, simulating conditions during orthodontic treatment, on the surface roughness of sound and demineralised enamel. This was designed as an in vitro preclinical safety assessment study, with the null hypotheses that there are no significant effects on the surface roughness of either sound or demineralised enamel following exposure to air polishing.

## 2. Materials and Methods

This in vitro preclinical safety assessment study was conducted and reported according to the guidelines for reporting pre-clinical in vitro studies on dental materials [25].

### 2.1. Collection and Preparation of Material

Fifty-four anonymised caries-free human molars were collected from the division of Oral surgery and Implantology at the University clinics of dental medicine in Geneva, Switzerland, and stored in distilled water at 25 °C in a constant-temperature incubator until further use. Each tooth was sectioned using a diamond wafering blade (Minitom Low-Speed Precision Cutting Machine STRUERS, Copenhagen, Denmark) and only the crowns were conserved.

The lingual enamel of each tooth crown was etched and bonded with composite resin on metal stubs (sandblasted and coated with silane—monobond). The composite resin was embedded in the tooth crown as flat as possible, with only the buccal enamel of the tooth exposed, to be polished and analysed. The buccal enamel initial surface integrity for all samples on the stubs was inspected and photographed using a high-resolution 3D digital microscope (Keyence 5000 VHX, Mechelen, Belgium).

For 42 samples, the superficial enamel layer was removed to create more consistent, reproducible surfaces using a water-cooled rotating polishing machine (Stationary micro grinder 400CS, Exakt, Histocom AG, Zug, Switzerland) with a sequential polishing protocol: 800-grit for 20 s, 1200-grit for 20 s, and 4000-grit for 45 s, followed by 3 min of ultrasonication to remove the smear layer on the enamel surface [26].

After this standardised polishing protocol, the 42 flattened and polished enamel samples were randomly assigned, using a random number generator in Microsoft Excel, into two experimental groups: *n* = 21 samples to be used as prepared (sound enamel), and *n* = 21 samples where artificial caries-like lesions were to be created (demineralised enamel).

**Figure 1:**
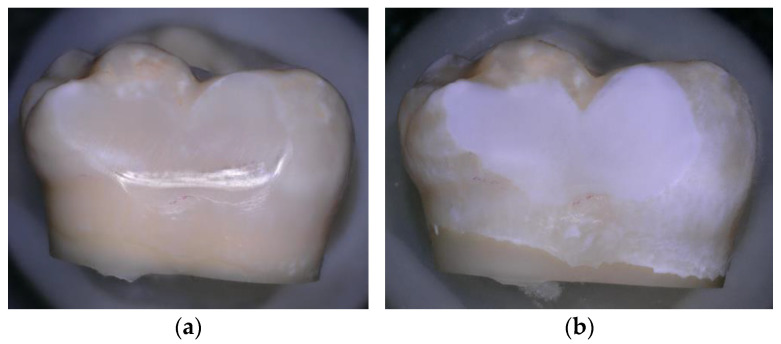
(**a**) Sound enamel before demineralisation; (**b**) Enamel after demineralisation, photographed using a confocal tandem scanning microscope (Keyence 5000 VHX digital microscope Belgium) with a 50× air objective in reflection scanning mode.

In order to induce artificial caries-like lesions on enamel for the demineralised enamel group, the following pH-cycling protocol was used: initial lesions were prepared on 21 enamel samples, immersed into 20 mL of demineralising solution, and contained in a small plastic bottle. The bottles were maintained at 25 °C in a constant-temperature incubator, without agitation. The demineralising solution was prepared from reagent grade chemicals and nominally contained 100 mmol/L lactic acid, 18.0 mmol/L calcium chloride, 7.8 mmol/L monobasic potassium phosphate, and 3 mmol/L sodium azide as a bacteriostat. The pH was adjusted to a pH of 4.3 with potassium hydroxide. In this fashion, subsurface enamel lesions were produced over a 2-week period, with the demineralising solution changed weekly. [27,28] (Figure 1).

### 2.2. Exposure of Samples to Air-Polishing Powders

The samples of sound enamel and demineralised enamel were each randomly divided into three experimental groups (*n* = 7), using a random number generator on Microsoft Excel. The three groups were exposed to different air-polishing powders, namely salt sodium bicarbonate 65 μm (EMS Air flow Classic); sugar alcohol erythritol 14 μm (EMS Air Flow PLUS); and a negative control (water without powder).

Each sample was first treated for 5 s with the respective powder using an AIRFLOW^®^ Prophylaxis Master clinical air-polishing unit (EMS, Nyon, Switzerland), and then retreated for 145 more seconds.

The duration of exposure to the respective powders was chosen based on the fact that, on average, 5 s corresponds to an instrumentation time per tooth surface sufficient to remove supragingival biofilm during supportive periodontal therapy [11,14,29] at each orthodontic appointment, and during a complete orthodontic treatment, it was estimated that, for a difficult treatment, a patient has approximately 30 appointments in total [30] where air polishing can be carried out, and thus 5 s at each appointment would equal 150 s of air polishing in total per tooth surface. Powder and water settings were standardised to 60% of the maximum level. Following a standardised clinical practice air-polishing technique, the nozzle of the AIRFLOW^®^ handpiece (EMS, Nyon, Switzerland) was kept in a constant circular motion, 4 mm away from the enamel surface with an angulation of 60° to the enamel target surface [4].

To guarantee constant powder flow and maximal efficacy, the powder chamber was refilled prior to each treatment session before and after instrumentation of each sample [31]. After each air-polishing procedure, the hose system was cleaned automatically of powder and water residues, and the enamel surfaces were cleaned under running water to remove powder residues, as in a clinical setting. After each air-polishing procedure, the samples were photographed using a high-resolution 3D digital microscope (Keyence 5000 VHX, Mechelen, Belgium).

### 2.3. Quantitative Surface Evaluation

The samples were stored in distilled water at 25 °C prior to testing. The surface topography of each sample was scanned using a non-contact confocal white light sensor profilometer generating 2D profiles (Cyberscan CT 100, cyberTECHNOLOGIES GmbH, Eching-Dietersheim, Germany), with 1 μm step-over distance and 3 nm vertical resolution. For the sound enamel, scanning was performed prior to surface air-abrasion, and after surface air-abrasion for 5 s and after 150 s. For the demineralised enamel, scanning was performed prior to pH-cycling, after pH-cycling, and after surface air-abrasion for 5 and 150 s.

A standard scan area (5 mm × 2 mm) was selected on the enamel sample surface as a target area. Screenshot photographs were taken for each sample and for each measurement procedure to replicate the same surface scan after pH-cycling and surface air-abrasion treatment. The resulting topographic images were analysed using surface metrology software (SCAN CT 8, cyberTECHNOLOGIES GmbH, Eching-Dietersheim, Germany) and for each sample the surface roughness (Ra—average surface roughness value) was measured.

Measurement of surface roughness and amplitude parameters was carried out using an optical white light probe with a z-resolution of 0.02 µm and scanning of x/y surfaces with a step of 1µm, on five lines of 5000 µm length every 200 µm on the target area. The average surface roughness was measured by the extraction of 5 profiles of 4000 µm length on the measured surface at a rate of 1 profile every 200 µm on the width of the target surface for each sample. The differences in the enamel surface roughness for each sample were calculated between pre-abrasion and post-abrasion, and between pre-pH cycling, post-pH-cycling, and post-abrasion, for 5 s and after 150 s.

Quantitative data were analysed using SPSS Statistics version 27 (SPSS, IBM, Armonk, New York, NY, USA). Two-way ANOVA tests were carried out to detect differences in surface roughness (independent variable) based on both time (0, 5, 150 s) and air-polishing treatment (negative control—water, erythritol powder, bicarbonate powder), followed by Tukey’s honest significance difference test as a post hoc test. These tests were performed independently for experiments on sound enamel and experiments on demineralised enamel. A *p*-value of <0.05 was considered statistically significant.

### 2.4. Qualitative Surface Evaluation

The qualitative assessment of air-flow powders and the enamel surface after polishing was evaluated using a Scanning electron microscope (SEM) (Sigma 300 VP, Zeiss, Jena, Germany).

- Assessment of Air flow powders:

SEM photomicrographs of bicarbonate (65 μm) and erythritol (14 μm) powders, at a magnification of 50×, 100×, 250×, 500×, and 1000×, were taken. Particle size distribution was randomly measured in one random area for 10 particles. In addition, chemical analysis of the powders was also performed, using Energy Dispersive X-ray Spectroscopy (EDX) with the Oxford AZTec Advanced Microanalysis system with an X-Max 50 ${\mathrm{mm}}^{2}$ detector with 127eV (@ Mn Ka) incorporated with the SEM.

- Assessment of enamel:

Changes in the surface characterisation of enamel due to air-polishing treatment were documented with the SEM, to evaluate the effects of air polishing on sound enamel (*n* = 6) and demineralised enamel (*n* = 6) without any previous flattening or polishing. The induced artificial caries-like lesions on enamel for the 6 samples were obtained by pH-cycling as described above.

The samples of sound enamel and demineralised enamel were divided in three experimental groups, namely salt sodium bicarbonate 65 μm; sugar alcohol erythritol 14 μm; and a negative control (water without powder).

Enamel (sound and demineralised) samples mounted on metal stubs embedded in composite with only the buccal enamel of the teeth exposed were gold sputter-coated with 20 nm (BT150 Bench Top Coaters HHV Ltd., Crawley, West Sussex UK). Subsequently, photomicrographs were taken of the surface enamel of each sample, prior to and after surface air-abrasion for each powder after 150 s, at magnifications of 13×, 34×, 100×, 200×, and 500×. Any disruption of the surface topography was interpreted as clinically significant [12].

- Power analysis

The sample size calculation, carried out on G*Power (Heinrich-Heine-Universität Düsseldorf, Germany), was based on a previous study [21], where the area-related arithmetic mean roughness value (Ra—average surface roughness values) of dentine surfaces after application of the powder jet were 0.917 ± 0.149 μm when using sodium bicarbonate (65 μm) and 0.648 ± 0.182 μm when using sugar alcohol erythritol (14 μm). The power was set at 80%, with an alpha (significance level) of 0.05. This resulted in a required sample size of *n*= 5 per group, and thus all groups included at least 5 teeth.

## 3. Results

### 3.1. Assessment of Air-Polishing Powders

**Figure 2:**
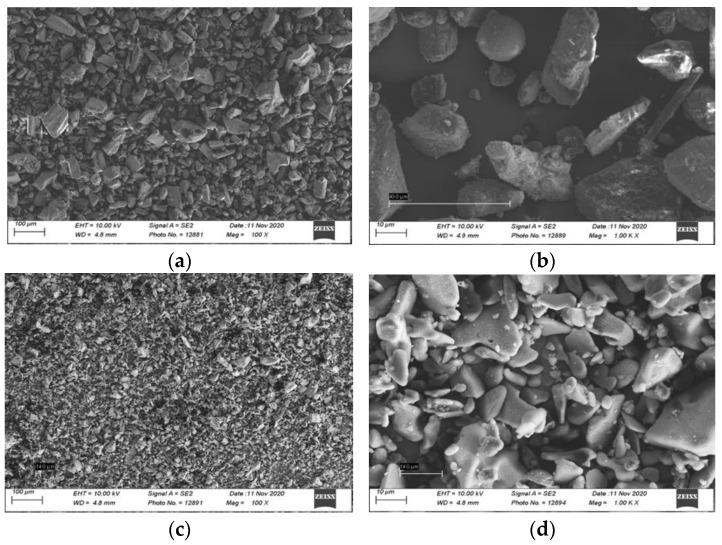
Scanning electron micrographs of air-polishing powder of salt sodium bicarbonate 65 μm (EMS Air flow Classic) powder under (**a**) 100× and (**b**) 1000× magnification; and sugar alcohol erythritol 14 μm (EMS Air Flow PLUS) powder under (**c**) 100× and (**d**) 1000× magnification.

- Particle size distribution:

Assessing the measurement of a random particle size under the SEM revealed a mean particle size of 49.45 ± 24.69 μm for bicarbonate powder, and 15.14 ± 3.18 μm for erythritol powder, confirming the smaller particle sizes for the erythritol air-polishing powder (Figure 2).

**Figure 3:**
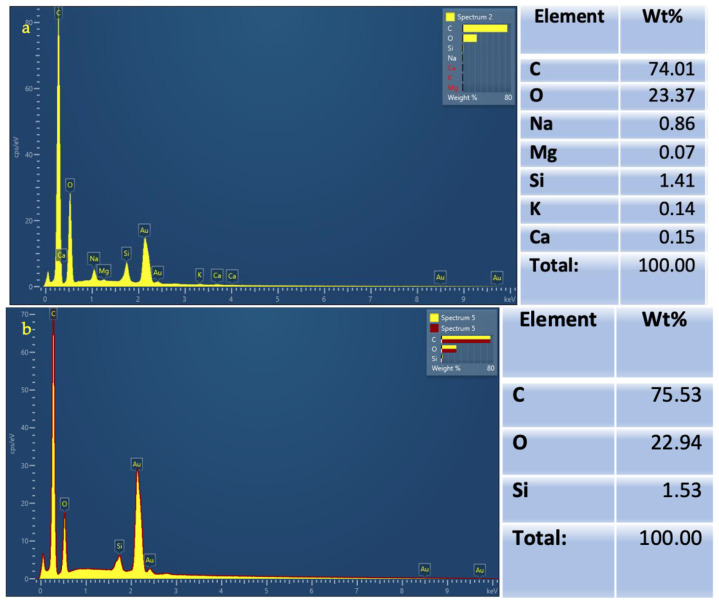
Chemical analysis using Energy Dispersive X-ray Spectroscopy (EDX) showing composition elements and their weight in percentages for (**a**) Salt sodium bicarbonate (EMS Air flow Classic) powder; (**b**) Sugar alcohol erythritol (EMS Air Flow PLUS) powder.

- Chemical composition elements and their proportional weight:

The chemical analysis (EDX) obtained for both powders is illustrated (Figure 3). The main elements composing the salt sodium bicarbonate powder were carbon, oxygen, and small percentages of sodium, magnesium, silicon, potassium, and calcium. The sugar alcohol erythritol composition was mainly carbon and oxygen, and a small percentage of silicon.

### 3.2. Quantitative Surface Evaluation

In order to ensure the homogeneity of the samples, the differences in the initial enamel surface roughness between the study groups were assessed, and no statistically significant differences were found.

- Sound enamel:

On sound enamel, the Ra was found to be roughly 0.17 ± 0.07 μm (in the control group). The treatment using water (control) produced no significant change in the Ra. Following treatment with air polishing on sound enamel (Table 1), the Ra was found to slightly increase after 5 s of air polishing with erythritol or bicarbonate powders, but this increase was not statistically significant. However, after 150 s of air polishing, the Ra increased with the erythritol treatment (by 0.28 μm), and even more so with the bicarbonate treatment (by 0.68 μm) (*p* < 0.01).

- Demineralised enamel:

On demineralised enamel, the Ra was found to be roughly 0.79 ± 0.56 μm (in the control group). The treatment using water (control) produced no significant change in the Ra. Following the air-polishing treatment on demineralised enamel (Table 1), the Ra was found to have already increased after 5 s of air-polishing treatment with both erythritol (1.03 μm) and bicarbonate powders (1.04 μm) (*p* = 0.025). This increase in the Ra was even more pronounced after 150 s of air polishing with both erythritol (2.48 μm) and bicarbonate powders similarly (2.49 μm) (*p* < 0.001).

### 3.3. Qualitative Surface Evaluation

**Figure 4:**
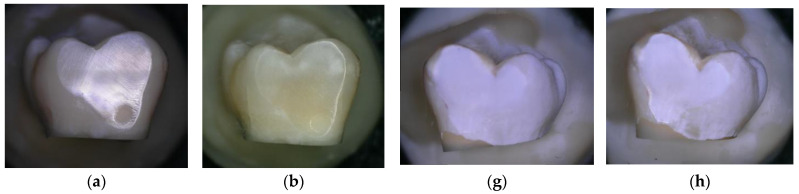

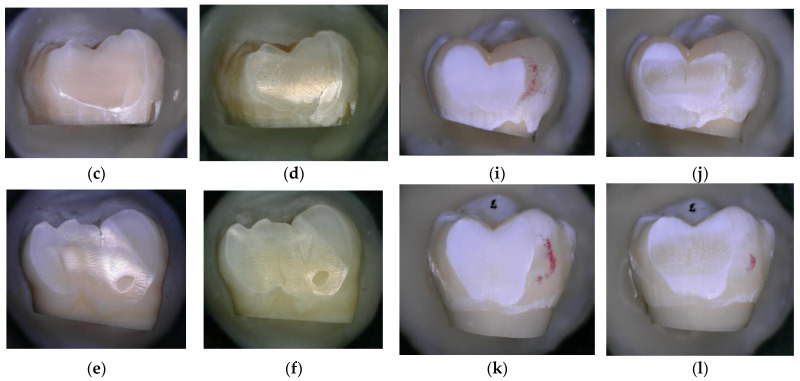
Enamel photographed using a high-resolution 3D digital microscope (Keyence 5000 VHX, at 50 magnification, before and after 150 s of treatment: (**a**) Sound enamel before and (**b**) after 150 s of treatment with water (negative control); (**c**) Sound enamel before and (**d**) after 150 s of treatment with powder; (**e**) Sound enamel before and (**f**) after 150 s of treatment with bicarbonate powder; (**g**) Demineralised enamel before and (**h**) after 150 s of treatment with water (negative control); (**i**) Demineralised enamel before and (**j**) after 150 s of treatment with powder; (**k**) Demineralised enamel before and (**l**) after 150 s of treatment with bicarbonate powder.

During each procedure, before and after air polishing, the samples were photographed using the 3D digital microscope for the qualitative assessment of the surface topography. Figure 4a,b shows no change in surface topography after treatment with the negative control (water). When comparing the surface of the sound enamel before and after the air-polishing treatment for 150 s using bicarbonate powder (Figure 4e,f), a greater visual surface roughness appears to be created than with the air-polishing treatment for 150 s using erythritol powder (Figure 4c,d). However, when comparing the surface of the demineralised enamel, both air-polishing treatments (using bicarbonate or erythritol) for 150 s seem to show an increase in visual surface abrasion and roughness, with an apparent removal of a portion of the demineralised enamel surface (Figure 4i–l).

**Figure 5:**
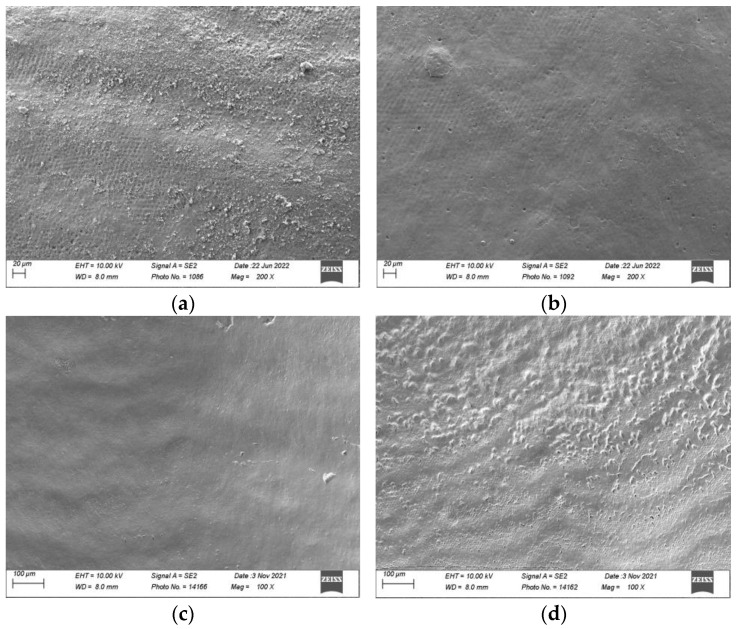

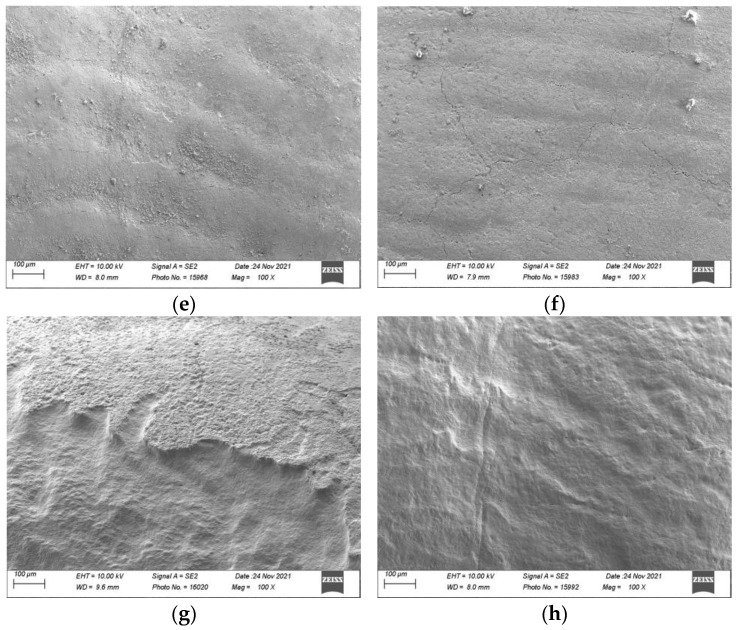
Scanning electron micrographs (Sigma 300 VP, Zeiss, Jena, Germany) of sound and demineralised enamel prior to and after 150 s of surface air-abrasion at a magnification of 100× and 200×: Sound enamel: (**a**) prior to treatment; (**b**) after 150 s of treatment with water (negative control); (**c**) after 150 s of treatment with erythritol powder; and (**d**) after 150 s of treatment with bicarbonate powder. Demineralised enamel: (**e**) prior to treatment; (**f**) after 150 s of treatment with water (negative control); (**g**) after 150 s of treatment with erythritol powder; and (**h**) after 150 s of treatment with bicarbonate powder.

The documentation from the SEM of the surface of the sound and demineralised enamel without any flattening or polishing, in the three experimental groups, is shown in Figure 5.

On sound enamel (Figure 5a–d), the water spray (negative control) seemed to remove the surface debris deposited on the enamel. The addition of air polishing with erythritol powder seemed to show changes to the surface topography (Ra had increased by 0.28 μm on the flattened and polished enamel), which was even more evident with the bicarbonate powder (Ra increased by 0.68 μm on the flattened and polished enamel).

On the demineralised enamel (Figure 5e–h) after 150 s of treatment, both erythritol (Figure 5g) and bicarbonate (Figure 5h) powders showed changes to the surface topography of the demineralised enamel (which was quantified on the flattened and polished enamel by an increase in Ra of 2.48 μm and 2.49 μm, respectively).

## 4. Discussion

**Figure 6:**
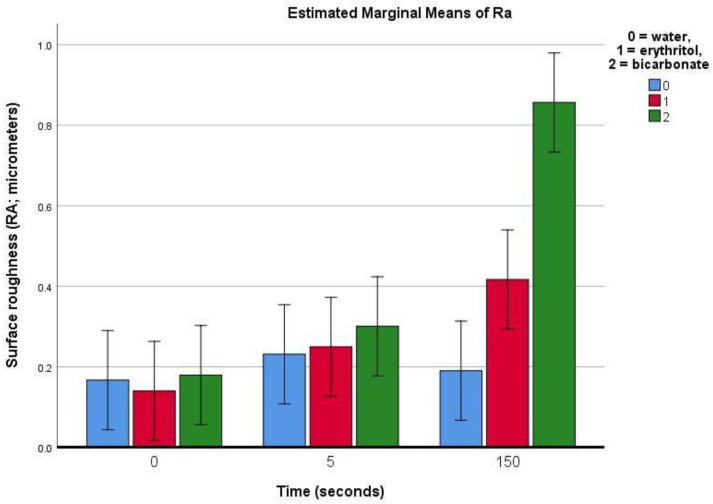
Bar chart showing the estimated means (bars) and 95% confidence intervals (whiskers) for the Ra (average surface roughness) for sound enamel prior to and after 5 or 150 s of surface air-abrasion using water (negative control), erythritol powder, or bicarbonate powder.

**Figure 7:**
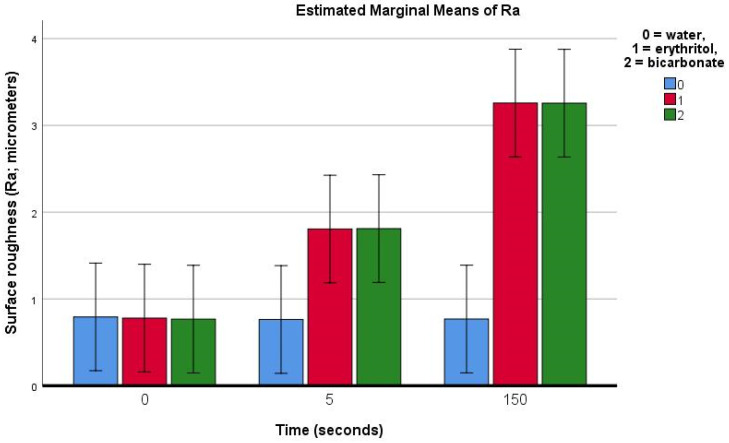
Bar chart showing the estimated means (bars) and 95% confidence intervals (whiskers) for the Ra (average surface roughness) for demineralised enamel prior to and after 5 or 150 s of surface air-abrasion using water (negative control), erythritol powder, or bicarbonate powder.

The present study aimed to evaluate the effects of different air-polishing powders (namely salt sodium bicarbonate and sugar alcohol erythritol) on the surface roughness of sound and demineralised human enamel, during repeated use equivalent to the duration of an orthodontic treatment. Our results showed an increase in surface roughness on sound enamel after 150 s of air polishing, which was more evident with the bicarbonate than with the erythritol treatment (Figure 6). On demineralised enamel, the increase in roughness was already evidenced with the profilometric measurements after 5 s of air-polishing treatment for both the erythritol and bicarbonate powders and this increase was even more pronounced after 150 s, similarly for both powders (Figure 7). The null hypotheses were thus rejected.

For supportive prophylactic therapy at each orthodontic appointment, a treatment interval of 5 s per tooth surface is accepted as sufficient to remove the supragingival biofilm [11,14,29] and for a complicated orthodontic treatment, it was estimated that approximately 30 appointments in total [30] are necessary to complete treatment, which is equal to an estimated 150 s of air-polishing treatment in total per tooth surface. This duration was thus chosen to illustrate the effects of air polishing. To make our results comparable, the settings recommended by the manufacture were used, and teeth exposed only to water without powder served as the negative control, thus, the results exclusively reflect the influence of the two powders and treatment times investigated.

This prophylaxis is important in order to avoid the development of white spot lesions, which is attributed to prolonged plaque accumulation around the brackets [32], and can be seen as early as four weeks after bracket placement, although the formation of caries usually takes at least six months [32,33]. Fixed appliances serve as plaque retention sites, and, in the absence of good oral hygiene, plaque accumulates and acidogenic bacteria causes marked demineralisation. These lesions (chalky white/brown) are commonly seen in the cervical and middle third of the buccal surface around the brackets, especially in the gingival region [34]. Tufekci et al. [35] observed a prevalence of white spot lesions of 38% six months into orthodontic treatment, and/or 46% twelve months into treatment, with at least one mild white spot lesion, and a few patients with moderate or severe demineralisation.

As a result, maintaining good oral hygiene during orthodontic treatment with regular professional oral hygiene is highly recommended, and prophylactic air polishing is an easy and efficient procedure, which does not necessitate the removal of archwires, ligatures, and springs and does not cause damage to the fixed appliances [9,10,36]. The effect on the integrity of the enamel surface, however, may vary depending on whether the enamel surface is intact [37], but little is known about the influence of prophylaxis with air polishing on demineralised tooth surfaces.

In an in vitro evaluation of wear by two prophylactic methods (sodium bicarbonate jet—Profident and pumice and brush) on bovine enamel (both sound and with artificial carious lesions), Honorio et al. [37] found higher wear for the demineralised enamel surfaces compared with the sound enamel, and the brush led to more wear when compared with the sodium bicarbonate jet. A direct comparison with our results, however, is not possible since we used human enamel, and the experimental setup was different.

Profilometry, which was used in the present study, is a simple and direct method that has the advantage of objective quantitative measurements of surface roughness without altering the surfaces of the samples. It requires a baseline flatness to obtain reliable measurements of any variation observed after the treatments [12,26], and thus the enamel surfaces we analysed were flattened. As the natural roughness of enamel is irregular, a standardised sequential polishing protocol was followed, to create more regularised and reproducible surfaces for the samples. With regards to the demineralised enamel surfaces, the pH-cycling protocol conducted in this study is a well-documented in vitro method to induce artificial caries-like lesions on enamel [27,28].

Specific regions of the enamel surface were analysed, and it has been previously shown that measuring one central cluster of unpolished and polished enamel is representative of the overall enamel surface roughness, before and after erosion. Polished enamel becomes significantly rougher after erosion and unpolished enamel becomes significantly smoother after erosion [38].

Increased surface roughness is a sign of surface damage. Surface damage resulting from kinetic abrasion is related to the particle size, hardness, and angularity of the abrasive powder [6,39]. Different studies have shown that smaller particles, such as glycine or erythritol, cause lesser damage to sound teeth or dental restorations [12,21]. Electron microscopy findings in the present study confirmed smaller particle sizes for erythritol powder compared with bicarbonate powder. Scanning electron microscopy images in the present study showed an obvious difference in the surface topography on sound enamel between erythritol and bicarbonate powders (Figure 5c,d). On the demineralised enamel surfaces, an evident change in surface topography could be visualised for both powders, evidenced by an abrasion of the surface of approximately 40 microns in depth (see Figure 5g,h). This abrasion of the surface was, however, not detailed in the present study as it was not one of the outcomes examined.

The chemical and physical conditions of the enamel surface change during the development of carious lesions, and thus, when performing any type of prophylactic treatment on demineralised surfaces, there is a greater chance of causing irreversible structural damage due to its lower resistance [37]. A qualitative evaluation of the enamel surface with scanning electron microscopy confirmed the profilometer-based findings of the present study, demonstrating greater apparent changes in surface roughness on demineralised enamel, regardless of the powder used.

A comparison of the present data with other studies proves difficult because previous studies often use non-human enamel, different powders, different air-polishing devices (with different air pressure, powder delivery mechanisms, or powder emission rates), different experimental setups, such as working distance and the angulation of the spraying nozzle, and different time intervals between powder applications. Different values of surface roughness and treatment outcomes are therefore incomparable because of this heterogeneity.

In the present study, the potential protective effects of saliva were not taken into consideration. This could have been an interesting experimental design to investigate, as has been undertaken in previous studies. Ribeiro et al. [40] evaluated the alterations of surface microhardness and wear caused by the sodium bicarbonate jet on sound bovine enamel and the further remineralising effect of artificial saliva. The sodium bicarbonate jet caused wear and a reduction in microhardness on the enamel surface and saliva promoted the recovery of initial surface microhardness and reduced wear, with this recovery seen best 24 h after treatment.

Arrais et al. [41] evaluated the effect of saliva in situ, in the recovery of the superficial structure of the demineralised tooth enamel on which a sodium bicarbonate jet was applied to the surface of the specimens simulating a prophylactic procedure. The effect of an additional mouth rinsing with a sodium fluoride solution was also evaluated. The performance of prophylaxis with the bicarbonate jet on enamel with artificial carious lesions promoted a loss of tooth structure, verified through the evaluation of wear and surface microhardness. The in-situ remineralisation promoted the recovery of part of the enamel structure lost, which was evidenced by a significant decrease in surface wear, although the surface microhardness value did not return to the value of healthy enamel. An additional rinse with the fluoride solution, right after prophylaxis, did not promote an increase in mineral gain in relation to the action of saliva alone.

Treating enamel surfaces mostly on cervical sites over several years with abrasive powder may cause substance loss and surface roughness, creating cumulative damage, especially on demineralised enamel surfaces. According to Bollen et al. [42], biofilm accumulation increases as the Ra increases and an Ra of 0.2 μm is the threshold surface roughness below which no impact on the bacterial retention could be expected. A vicious cycle may occur, whereby plaque accumulation increases the risk of caries, gingival irritation, and periodontal inflammation [32], and air polishing removes the surface plaque but also creates irreversible structural damages on demineralised enamel and increases the surface roughness above the threshold, increasing bacterial retention.

The present study has several limitations. Previous studies have suggested that natural enamel is less susceptible to the effects of acid-induced erosion compared with polished enamel [43]. Polished enamel was used in our study, however, in order to make our experimental setup more homogeneous and reproducible. Daily toothbrushing can also influence the abrasion of demineralised enamel, and studies have found a significant loss of the acid-softened enamel surfaces [44], mostly with conventional rotating–oscillating toothbrushing [45], increasing the surface roughness and wear. This is an important factor that was not investigated in the present study but should be considered in a clinical setting.

No saliva exposure was used in this study, which is different from the real-world situation where teeth are constantly bathed in saliva, which may offer a repair mechanism in between treatment sessions with air-polishing prophylaxis. Saliva is rich in minerals and proteins, it is supersaturated with calcium and phosphate ions, and it lubricates the teeth. Thus, saliva acts against demineralisation and may be able to recover the slight mineral loss of enamel caused by prophylaxis using air polishing. Furthermore, fluoride can increase the rate of re-mineralisation due to its mechanism of action [46].

Finally, although surface roughness is a very important parameter to investigate, another parameter which could be assessed and quantified is the loss of volume in the enamel defects, which could be cumulative after repeated exposure to air-polishing powders. The amount of surface volume loss remains to be investigated. With a mean value of 1.14 mm of enamel thickness on the buccal aspect of human maxillary central incisors [47], the loss of tooth surface, especially on demineralised enamel, would be interesting to assess.

Under the premise that the brushes are applied for the same time interval and similar brushing technique and force, the results of this investigation indicate that enamel loss after acidic attack may be increased by using certain power or sonic toothbrushes compared with the tested manual brush [48].

Air polishing as a prophylactic technique may present minimal adverse effects on surface roughness but implementing it as a preventive measure to prevent demineralisation or initial caries, which may lead to more significant changes in surface roughness and volume loss in a short period of time, probably outweighs any potential disadvantage.

## 5. Conclusions

An increase in surface roughness is observed on sound enamel after 150 s of air polishing, more so with bicarbonate than with erythritol treatment, but this is probably clinically irrelevant. On white spot lesions, however, one should be aware that the surface roughness of enamel will significantly increase with both erythritol and bicarbonate air-polishing powders, especially with longer exposure times.

## Figures and Tables

**Table 1:** Mean and standard deviation (SD) of Ra (average surface roughness) for sound and demineralised enamel prior to and after 5 or 150 s of surface air-abrasion using water (negative control), erythritol powder, or bicarbonate powder.

Group	Powder	Ra (µm) before Treatment (mean ± SD)	Ra (µm) after 5 s of Treatment (mean ± SD)	Ra (µm) after 150 s of Treatment (mean ± SD)
Sound Enamel	Water (Control)	0.17 ± 0.07 ^a^	0.23 ± 0.08 ^a^	0.19 ± 0.06 ^a^
	Erythritol	0.14 ± 0.04 ^a^	0.25 ± 0.02 ^a^	0.42 ± 0.07 ^b^
	Bicarbonate	0.18 ± 0.07 ^a^	0.30 ± 0.09 ^a^	0.86 ± 0.45 ^c^
Demineralised Enamel	Water (Control)	0.79 ± 0.56 ^a^	0.76 ± 0.42 ^a^	0.77 ± 0.36 ^a^
	Erythritol	0.78 ± 0.56 ^a^	1.81 ± 1.08 ^b^	3.26 ± 1.57 ^c^
	Bicarbonate	0.77 ± 0.44 ^a^	1.81 ± 0.68 ^b^	3.26 ± 0.90 ^c^

Legend: different letters denote statistically significant differences between groups in the experiments on sound enamel and in the experiments on demineralised enamel. No comparisons are made between results on sound enamel and demineralised enamel.

## Data Availability

The data presented in this study are available on request from the corresponding author.
